# Novel Rapid Test for Detecting Carbapenemase

**DOI:** 10.3201/eid2604.181655

**Published:** 2020-04

**Authors:** Yanfang Feng, Akilan Palanisami, Jerrin Kuriakose, Michael Pigula, Shoaib Ashraf, Tayyaba Hasan

**Affiliations:** Massachusetts General Hospital and Harvard Medical School, Boston, Massachusetts, USA (Y. Feng, A. Palanisami, J. Kuriakose, M. Pigula, S. Ashraf, T. Hasan);; Harvard-MIT Health Sciences and Technology, Cambridge, Massachusetts, USA (T. Hasan)

**Keywords:** Laboratory diagnosis, carbapenemase, gram-negative bacteria, fluorescence, bacteria, rapid test

## Abstract

We developed a carbapenemase test based on the ability of imipenem to inhibit noncarbapenemase β-lactamases. The test uses bacterial isolates with a fluorescent β-lactamase substrate, producing objective results with 100% sensitivity and specificity in 10 minutes. The assay is inexpensive and consists of only 1 mixing step.

As a potent β-lactamase, carbapenemase can degrade almost all β-lactam antimicrobial drugs, including the carbapenems, regarded as the last line of therapy for many life-threatening infections (*1*,*2*). Various epidemic types of carbapenemase have been reported globally, including *Klebsiella pneumoniae* carbapenemase, Verona integron-encoded metallo-β-lactamase, *Serratia marcescens* enzyme, imipenem-hydrolyzing β-lactamase, New Delhi metallo-β-lactamase, oxacillinase, metallo-β-lactamase, and São Paulo metallo-β-lactamase (*1*). If uncontrolled, the spread of these carbapenemases is expected to increase therapeutic failure and leave many patients with no effective treatment options.

Despite the urgency, timely carbapenemase detection remains a challenge for microbiology laboratories. Phenotypic assays are inexpensive and easily performed, but their use requires 24–48 hours and many lack sensitivity or specificity (*3*). The widespread use of other assays (e.g., molecular tests of carbapenemase genes, mass spectrometry detection of carbapenem hydrolysis) is impeded by the expertise required to perform them and their cost (*4*,*5*). The recently developed (2012) Carba NP test and variants are elegant solutions, but their use requires up to 2 hours (*6*). Further improvements in test rapidity and simplicity are highly desirable, especially for patients in critical condition, who need immediate therapy and infection control action.

We demonstrate that by using fluorescence identification of β-lactamase activity (FIBA), carbapenemase production in bacteria can be detected sensitively and specifically in 10 minutes, with only 1 step. FIBA uses a dark fluorescence probe, β-LEAF (β-lactamase enzyme–activated fluorophore), which turns fluorescent when cleaved by β-lactamases, including penicillinases, extended-spectrum β-lactamases (ESBL), AmpC β-lactamases, and carbapenemases (*7**,**8*). Thus, the rate of fluorescence increase (hereafter called increase rate) is a measure of the bacterial β-lactamase activity and is reduced as the β-lactamase activity is hampered. For a noncarbapenemase β-lactamase, the increase rate will be reduced by the addition of imipenem, which binds the enzyme active site and blocks β-LEAF access (*2*). In contrast, the increase rate for a carbapenemase is relatively unaffected by imipenem addition because carbapenemase is able to rapidly cleave the imipenem and relieve the inhibition (*1*). Accordingly, bacteria that produce carbapenemases can be detected by comparing the increase rate with and without imipenem (Figure; Appendix Figure 1).

**Figure:**
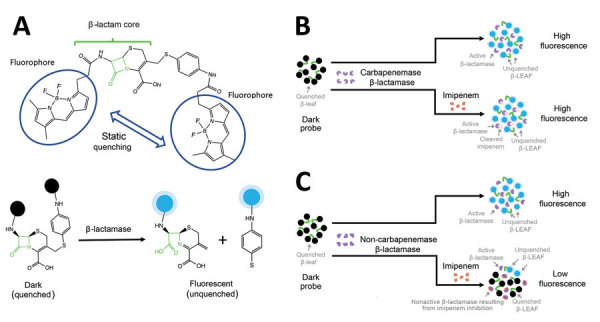
Schematic illustration of the principle of fluorescence identification of β-lactamase activity. A). The β-lactamase–activated fluorophore probe comprises a cleavable β-lactam core conjugated to 2 fluorophores (circled) that are quenched because of close proximity. This construct was designed to mimic the enzymatic degradation properties of easily cleavable β-lactam antimicrobial drugs. When this probe is attacked by β-lactamase, the probe core is cleaved, leading to the separation of the fluorophores and the recovery of their fluorescent properties (fluorescent state). B) Assay profile for carbapenemase-producing bacteria. C) Assay profile for non–carbapenemase-producing bacteria. Black, quenched fluorophore; blue, unquenched fluorophore turning fluorescent; green, β-lactam core; red, imipenem; purple, β-lactamase. β-LEAF, β-lactamase enzyme–activated fluorophore.

FIBA is performed in a 96-well plate. Each well contains 50 μL of 20 µmol/L β-LEAF, 25 μL of phosphate-buffered saline with or without 40 μmol/L imipenem (Cayman Chemical, https://www.caymanchem.com), and 10 μL of either 1 mg/mL polymyxin B nonapeptide or 1% 3-[(3-cholamidopropyl)dimethylammonio]-1-propanesulfonate (Sigma-Aldrich, https://www.sigmaaldrich.com), which act as weak or strong permeabilizers, respectively. To start the assay, 25 μL of 1 × 10^10^ CFU/mL bacterial suspension made by colonies grown overnight on BHI agar (Sigma-Aldrich) is added to each well. To monitor the increase rate, fluorescence measurement is then performed at 37°C at 10-s intervals for 10 min with Ex/Em 450/510 nm in the plate reader (Spectramax M5 plate reader, Molecular Devices, https://www.moleculardevices.com). For each bacterial sample, we performed the reactions in duplicate and averaged the results. We objectively interpreted the fluorescence measurements by using an automated Python script (Appendix), which required a few seconds after assay completion.

We tested FIBA on 76 randomly selected infection isolates from either the Centers for Disease Control and Prevention (*9*) or the American Type Culture Collection (https://www.atcc.org). The MICs of these isolates, if not predetermined, were measured by the 2017 Clinical Laboratory and Standards Institute (https://clsi.org) broth dilution method. Genetic test results for β-lactam resistance were provided with the isolates. Among these, 55 were carbapenemase positive, carrying the major epidemic carbapenemase types including *K. pneumoniae* carbapenemase (n = 20), imipenem-hydrolyzing β-lactamase (n = 2), metallo-β-lactamase (n = 4), New Delhi metallo-β-lactamase (n = 10), oxacillinase (n = 8), *S. marcescens* enzyme (n = 2), São Paulo metallo-β-lactamase (n = 1), Verona integron-encoded metallo-β-lactamase (n = 6), and New Delhi metallo-β-lactamase oxacillinase (n = 2). The other 21 isolates expressed noncarbapenemase β-lactamases, which involved 9 isolates with only ESBL, 3 isolates with both ESBL and porin modification, 6 isolates with only AmpC β-lactamase, and 3 isolates with both ESBL and AmpC β-lactamase. Among these isolates, 3 were carbapenem resistant. The entire panel, which included 28 colistin-resistant strains (MIC >4 ug/mL), was classified successfully with FIBA (Appendix Tables 1, 2), resulting in 100% sensitivity (95% CI 94%−100%) and 100% specificity (95% CI 84%−100%). 

The primary limitation of this study is the small number of isolates evaluated. However, the breadth of isolates studied here included 8 enzyme types across 16 species, suggesting the generality of the approach.

FIBA can be performed ≈10 times faster than the most rapid carbapenemase test commercially available while maintaining comparable sensitivity and specificity (*6*,*10*). Its automated analysis improves turnaround time and reduces operator variability. With a reagent cost/assay of ≈US $1, FIBA is close in price to phenotypic tests but substantially faster and less labor intensive. Furthermore, the FIBA paradigm is extensible; by replacing imipenem with other known subtype-dependent inhibitors of carbapenemase (e.g., clavulanic acid, EDTA), rapid carbapenemase subtyping may also be possible. Our study demonstrates that low-cost, rapid assessment of carbapenemase can be performed in a 1-step format suitable for large-scale epidemiologic studies, thereby providing a new tool for infection outbreak control.

Appendix Supplementary methods and results for study of novel rapid test for detecting carbapenemase.
