# Supplementary material for: Novel Rapid Test for Detecting Carbapenemase
Source: Emerg Infect Dis. 2020 Apr;26(4):793–5. doi: 10.3201/eid2604.181655 (PMC7101118; doi:10.3201/eid2604.181655)
Supplement: Appendix — Supplementary methods and results for study of novel rapid test for detecting carbapenemase. [file 18-1655-Techapp-s1.pdf]

Article DOI: https://doi.org/10.3201/eid2604.181655

Novel Rapid Test for Detecting Carbapenemase

Appendix

Supplementary Methods

The imipenem solution was prepared and used within 30 mins (stored at 4 °C during this time). Samples were physically tested in a blind and random fashion. To develop the subsequent analysis, the data were unblinded to determine the appropriate analysis thresholds. The FIBA limit of detection was $10^{9\text{-}10}$ CFU/ml for this panel.

The automated Python analysis for FIBA test

To quickly determine carbapenemase activity from the fluorescence time course data, a python script was developed to easily analyze the excel spreadsheet files generated by the fluorescence plate reader. The analysis is as follows: To quantify the changes of the β-lactamase activity upon the addition of imipenem, a β-lactamase inhibition index (**BI,** see equation **II**), defined as the ratio of fluorescence increase rate (**R,** see equation **I**) between wells without and with imipenem, was created. **BI** increases with imipenem inhibition (non-carbapenemase β-lactamase behavior), and an isolate with **BI**≤0 is classified as carbapenemase-positive.

Among the samples with **BI**>0 in the presence of 100 µg/ml cell membrane permeabilizer polymyxin B nonapeptide (PMBN), a parallel assay with another permeabilizer, 0.1% 3-[(3-cholamidopropyl)dimethylammonio]-1-propanesulfonate (CHAPS), was also performed. This is to rule out false negatives that may be caused by insufficient permeabilization due to bacterial polymyxin resistance. Isolates with a **BI**≤0 under the condition of CHAPS were also classified as carbapenemase-positive. All remaining isolates were classified as carbapenemase-negative.

The equations used in Python analysis:

I.

$$\mathbf{R} = \sum_{i=1}^{N} v_i$$

where v is the fluorescence increase per time step, i is the time index of the sorted v (i.e., v_1 is the largest fluorescent difference, v_2 is the second largest fluorescent difference, etc.) and N is the number of v to be summed. v was determined from a 50 second running average of the fluorescent time course, and N was set to 45.

II.

$$\boldsymbol{BI} = \left(\sum_{i=1}^{N} v_i^{0\ \mu M} \Big/ \sum_{i=1}^{N} v_i^{10\ \mu M} \right) - \mathrm{BI}_0$$

where v_i^C is the fluorescence increase per time step at imipenem concentration C , and BI_0, designated as the **BI** cut-off value for the enzyme inhibition, is 1.25.

Appendix Table 1. Carbapenemase-producing isolates subjected to the FIBA* test

Carbapenemase category					Carbapenem susceptibility‡				FIBA test
Ambler class	Type	Subtype	Species	No.†	Imi	Mer	Ert	Dor	result§
A	KPC	KPC-2	*C.freundii*	1	16	> 8	> 8	> 8	+
			E.cloacae	1	8	8	> 8	4	+
			K.pneumoniae	1	> 8	16	≤ 0.25	> 8	+
			M.morganii	1	8	4	8	4	+
			P.mirabilis	1	16	0.5	1	4	+
		KPC-3	*E.cloacae*	2	≥8	4-8	0.5	1- > 8	+
			E.coli	2	4-8	4	0.5 - 8	≤ 0.25 - 4	+
			K.ascorbata	1	4	8	8	4	+
			K.oxytoca	1	4	1	0.5	0.5	+
			K.ozaenae	1	> 8	> 64	0.5	> 8	+
			K.pneumoniae	3	> 8	> 8	≥ 4	> 8	+
			R.ornithinolytica	1	4	1	1	2	+
		KPC-4	*E.cloacae*	1	0.5	1	0.5	> 8	+
		KPC-5	*P.aeruginosa*	1	> 8	> 64	1	> 8	+
		KPC-6	*E.cloacae*	1	4	4	0.5	8	+
			P.mirabilis	1	16	2	2	2	+
	SME	SME-3	*S.marcescens*	2	> 8	> 64	4	≤ 0.25- 0.5	+
	NMC-A	NMC-A	*E.cloacae*	2	≥ 32	> 8	> 8	> 8	+
B	NDM	NDM-1	*E.coli*	1	8	> 16	> 8	> 8	+
			K.pneumoniae	2	> 8	> 8	1 - > 8	> 8	+
			M.morganii	1	2	8	4	> 8	+
			P.mirabilis	1	32	4	4	> 8	+
			P.rettgeri	1	8	32	4	≤ 0.25	+
			S.senftenberg	1	4	8	> 8	8	+
			A.baumannii	1	> 8	64	1	> 8	+
			Citrobacter spp.	1	16	> 8	> 8	> 8	+
			E.cloacae	1	4	32	≤ 0.25	> 8	+
		NDM-1/OXA-64	*A.baumannii*	1	> 8	64	0.5	> 8	+
		NDM-6	*E.coli*	1	16	> 8	> 8	> 8	+
	VIM	VIM-1	*E.cloacae*	1	4	2	2	4	+
			K.pneumoniae	1	4	4	1	4	+
		VIM-2	*P.aeruginosa*	2	> 64	> 8	> 8	> 8	+
		VIM-27	*K.pneumoniae*	1	64	> 8	> 8	> 8	+
		VIM-4	*P.aeruginosa*	1	> 64	> 8	> 8	4	+
	IMP	IMP-1	*P.aeruginosa*	1	> 64	> 8	> 8	> 8	+
		IMP-14	*P.aeruginosa*	1	64	> 8	> 8	> 8	+
		IMP-4	*K.pneumoniae*	2	1 - 4	2 - 4	2 - 4	4 - 8	+

Carbapenemase category			Species	No.†	Carbapenem susceptibility‡				FIBA test result§
Ambler class	Type	Subtype			Imi	Mer	Ert	Dor	
	SPM	SPM-1	*P.aeruginosa*	1	> 64	> 8	> 8	> 8	+
D	OXA	OXA-48	*E.aerogenes*	1	4	2	2	2	+
			K.pneumoniae	1	4	8	> 8	8	+
		OXA-58/100	*A.baumannii*	2	16 - 32	> 8	> 8	8	+
		OXA-66/72	*A.baumannii*	1	> 64	> 8	> 8	> 8	+
		OXA-181	*K.ozaenae*	1	4	4	> 8	4	+
			K.pneumoniae	1	2	4	> 8	4	+
		OXA-232	*K.pneumoniae*	1	4	> 8	> 8	> 8	+

*FIBA, Fluorescence identification of β-Lactamase activity.
†No., number of isolates tested.
‡MIC of the tested isolates for doripenem (Dor), ertapenem(Ert), imipenem(Imi) and meropenem(Mer).
§FIBA test result: -, negative; +, positive. All the results shown here were based on the average of two independent replicates. With the permeabilizer PMBN, there are 4 out of 57 (7%) isolates labeled as false negatives which are subsequently found positive with the permeabilizer CHAPS.

Appendix Table 2. Non-carbapenemase-producing isolates subjected to the FIBA* test

β-Lactamase category		Species	No.†	Carbapenem susceptibility‡				FIBA test result§
Type	Subtype			Imi	Mer	Ert	Dor	
ESBL	CTX-M-14, TEM-1B	*E.coli*	1	≤ 0.5	≤ 0.12	1	≤ 0.12	-
	CTX-M-15, SHV-1, TEM-1B, OXA-1/10¶	*K.pneumoniae*	1	1	2	> 8	2	-
	CTX-M-2, SHV-83, TEM-1A, OXA-9/10¶	*K.pneumoniae*	1	8	> 8	> 8	> 8	-
	SHV-3	*E.coli*	1	≤ 0.5	≤ 0.12	≤ 0.12	≤ 0.12	-
	SHV-4	*E.coli*	1	≤ 0.5	≤ 0.12	≤ 0.12	≤ 0.12	-
	SHV-12	*K.pneumoniae*	1	≤ 0.5	2	> 8	1	-
	SHV-12¶	*K.pneumoniae*	1	≤ 0.5	≤ 0.12	0.25	≤ 0.12	-
	TEM-3	*E.coli*	1	≤ 0.5	≤ 0.12	≤ 0.12	≤ 0.12	-
	TEM-10	*E.coli*	1	≤ 0.5	≤ 0.12	≤ 0.12	≤ 0.12	-
	TEM-12	*E.coli*	1	≤ 0.5	≤ 0.12	≤ 0.12	≤ 0.12	-
	TEM-26	*E.coli*	1	≤ 0.5	≤ 0.12	≤ 0.12	≤ 0.12	-
	TEM-52B	*E.coli*	1	≤ 0.5	0.25	1	0.25	-
AmpC	ACT-7	*E.cloacae*	1	≤ 0.5	≤ 0.12	0.25	≤ 0.12	-
	ACT-15	*E.cloacae*	1	≤ 0.5	≤ 0.12	1	≤ 0.12	-
	CMY-2	*E.coli*	2	≤ 0.5	≤ 0.12 - 1	≤ 0.12 - 2	≤ 0.12	-
	cAmpC	*E.aerogenes*	1	≤ 0.5	≤ 0.12	1	≤ 0.12	-
	cAmpC	*E.cloacae*	1	≤ 0.5	≤ 0.12	≤ 0.12	≤ 0.12	-
ESBL& AmpC	CTX-M14; DHA-1, SHV-11, TEM-1B¶	*K.pneumoniae*	1	16	8	> 8	8	-
	cAmpC, TEM-1B	*E.cloacae*	1	≤ 0.5	≤ 0.12	1	≤ 0.12	-
	CMY-2, TEM-1B	*E.coli*	1	≤ 0.5	≤ 0.12	≤ 0.12	≤ 0.12	-

*FIBA, fluorescence identification of β-Lactamase activity.
†No., number of isolates tested.
‡MIC of the tested isolates for doripenem (Dor), ertapenem(Ert), imipenem(Imi) and meropenem(Mer).
§FIBA test result: -, negative; +, positive.
¶Porin modifications were present together with β-lactamase. All the results shown here were based on the average of two independent replicates.

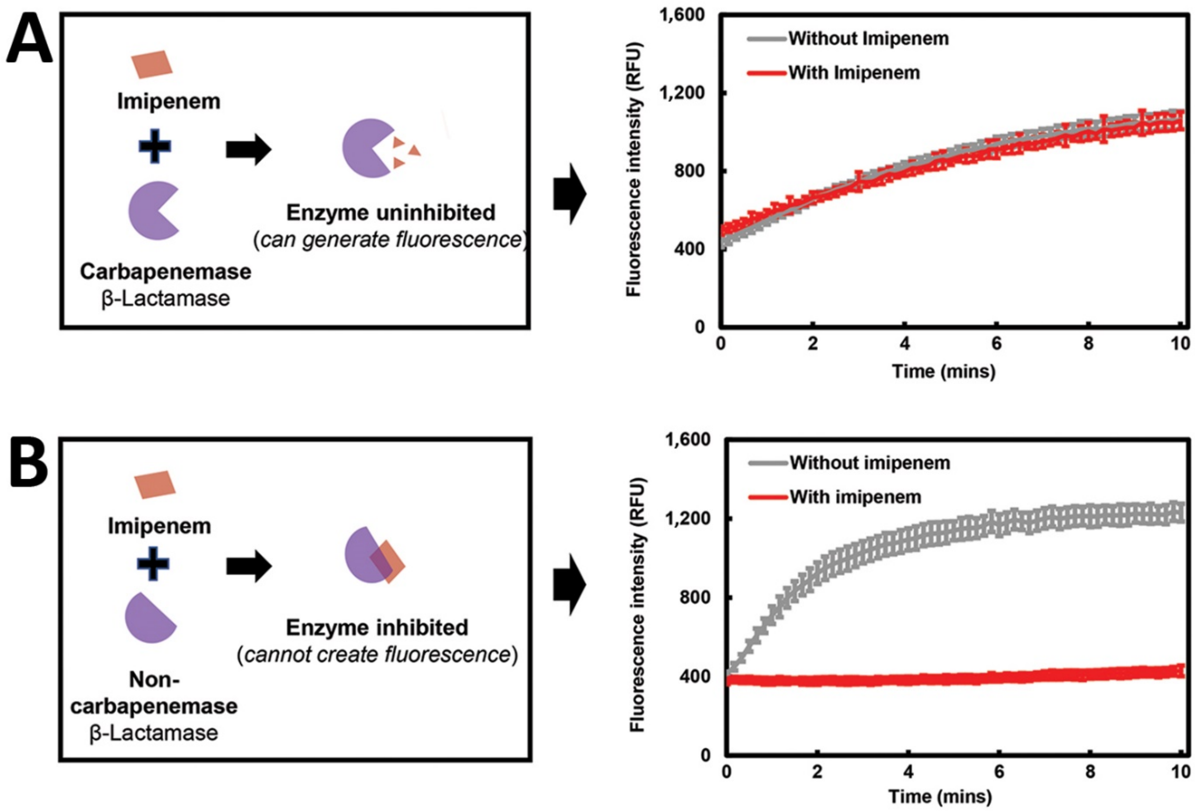

Appendix Figure 1. The detection of bacterial carbapenemase production by the fluorescence identification of β-lactamase activity. A) Carbapenemase producing isolates cleave β-lactamase enzyme-activated fluorophore irrespective of imipenem addition, as exemplified here by the strain # 0147 from the CDC isolate bank. B) Non-carbapenemase β-lactamases are unable to cleave β-lactamase enzyme-activated fluorophore when inhibited by imipenem, as shown here by the isolate # 0065 from the CDC isolate bank.

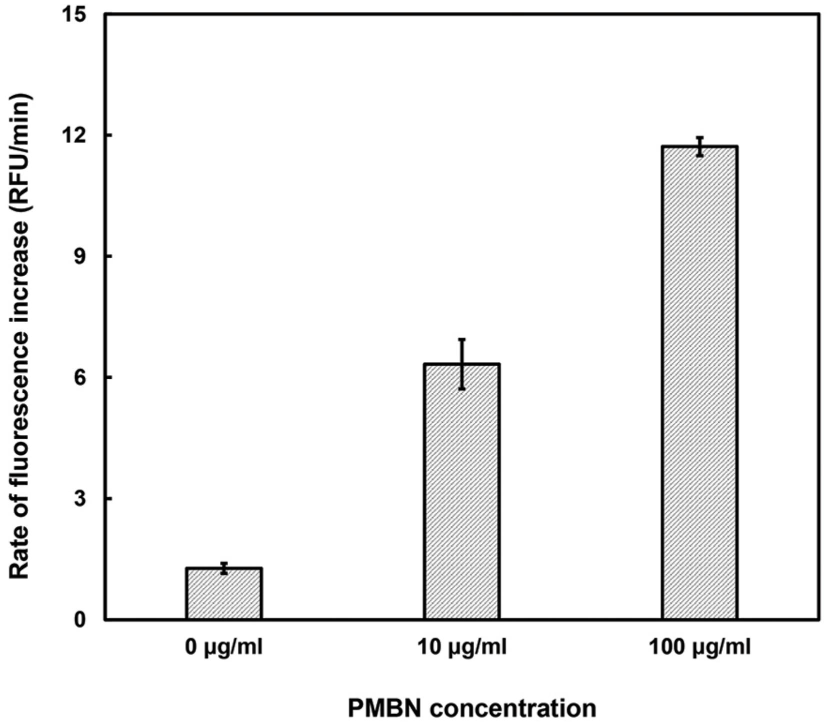

Appendix Figure 2. The rate of fluorescence increase in FIBA* increases with the addition of PMBN†. *FIBA, fluorescence identification of β-lactamase activity; †PMBN, polymyxin B nonapeptide; The strain used here as an illustration is a β-lactamase producing strain from ATCC (*Escherichia coli*, ATCC® BAA-196™).
